# Living with Dysphagia and Dysarthria: A Qualitative Exploration of the Perspectives of People with Motor Neuron Disease and Their Caregivers

**DOI:** 10.3390/healthcare13182306

**Published:** 2025-09-15

**Authors:** Rebecca Packer, Anna Rumbach, Anna Farrell, Nicole Hutchinson, Stacey Verner-Wren, Robert Henderson, Pamela McCombe

**Affiliations:** 1 School of Health and Rehabilitation Sciences, The University of Queensland, St Lucia 4072, Australia; 2 Centre for Motor Neuron Disease Research, The University of Queensland, St Lucia 4072, Australia; 3 Princess Alexandra Hospital, Metro South Health, Wooloongabba 4102, Australia; 4 Royal Brisbane and Women’s Hospital and Community Palliative Care, Metro North Health, Chermside 4032, Australia; 5 Prince Charles Hospital, Metro North Health, Chermside 4032, Australia

**Keywords:** amyotrophic lateral sclerosis, caregivers, deglutition disorders, dysarthria, dysphagia, motor neuron disease, motor speech disorders, qualitative research

## Abstract

**Background/Objectives**: Dysphagia and dysarthria are common and distressing symptoms of motor neuron disease (MND) progression. The medical complications of dysphagia and the influence of dysarthria on communication effectiveness have been documented. The aim of the current study was to describe the lived experience of dysphagia and dysarthria from the perspectives of both people with motor neuron disease (pwMND) and their caregivers. **Methods**: A qualitative descriptive study influenced by phenomenological principles was utilized in interviews with six pwMND and six caregivers. **Results**: Three themes were developed that captured participants’ experiences of dysphagia and dysarthria: (1) “This is the way things are”; (2) Your whole life changes, but some things stay the same; (3) Juxtaposition to information and support. **Conclusions**: The findings advance our understanding of the lived experience of dysphagia and dysarthria in MND. Health professionals need to consider broader assessment practices across both mealtimes and communicative interactions and each individual’s unique information and support needs when providing healthcare information.

## 1. Introduction

Motor neuron disease (MND) is a progressive, neurological, life-limiting, and multi-system disease that has significant medical [1], psychosocial, and quality-of-life impacts on people diagnosed with the disease [2]. The effects of MND are diverse, affecting an individual’s ability to move, breathe, speak, and swallow [1]. In addition, there is growing awareness that MND can lead to executive deficits, resulting in cognitive, behavioral, and emotional changes [1]. Among the most distressing symptoms are dysphagia and dysarthria, which affect up to 80% and 95% of people with MND (pwMND), respectively [3,4,5]. Dysphagia (i.e., difficulty swallowing) is typically oropharyngeal, involving impaired tongue control, pharyngeal contraction, and reflex timing. Though sensory changes in swallowing have been reported, the evidence remains inconclusive [6]. Dysarthria (i.e., a motor speech disorder), often spastic–flaccid in nature, results in slow, effortful speech, imprecise articulation, and voice changes [3].

Research to date has primarily focused on the clinical aspects of dysphagia and dysarthria, such as aspiration risk, enteral nutrition, and communication effectiveness [7,8]. Whilst previous studies have linked dysphagia and dysarthria to psychological distress, few have explored the practical and psychosocial impacts of these impairments on daily life. In addition, dysphagia and dysarthria symptoms in pwMND also have significant implications for those who care for them. Informal caregivers are often responsible for managing mealtimes, facilitating communication, and providing emotional support; roles that can be both physically and emotionally demanding. As such, understanding the caregiver experience is essential to fully grasp the broader impact of dysphagia and dysarthria in MND. It is established that informal caregivers play a central role in the symptomatic management of MND, and the substantial burden of caregiving in MND is well recognized [9,10] and correlated with the level of physical functioning of the patient [10].

To improve supportive care, it is essential to understand how pwMND and their caregivers experience and adapt to swallowing and speech impairments. Preliminary research has begun to document experiences of dysphagia and dysarthria separately. Two qualitative studies conducted by Lisiecka and colleagues, using interpretive phenomenological analysis, explored experiences of dysphagia from the perspective of pwMND and caregivers [11,12]. Themes included living in the present, self-management, and the transformation of mealtime experiences. Notably, some participants reported that dysarthria had a greater impact on daily life than dysphagia, despite not being the focus of the study [11].

The lived experiences of dysarthria in MND are poorly described in the literature. However, Olmstead and colleagues surveyed pwMND and caregivers about communication challenges, revealing widespread difficulty with intelligibility and interest in supportive interventions [13]. Paynter and colleagues conducted interviews with pwMND and caregivers regarding their experiences of healthcare in the context of communication impairment [14]. The results of this study highlighted the impact of communication impairment on healthcare interactions and the effort needed by pwMND and their caregivers to maximize involvement in their healthcare. Whilst these findings provided important insights, the study did not explore the experiences of pwMND and their caregivers in day-to-day life.

These studies have provided important preliminary insights into the experiences of dysphagia and dysarthria from the perspectives of pwMND and caregivers. To better support pwMND and their families, we must further elucidate how they experience and cope with swallowing and communication impairments in daily life. Therefore, the aim of the current study was to explore how pwMND and their caregivers experience, adapt to, and manage the challenges of dysphagia and dysarthria in daily life.

## 2. Materials and Methods

### 2.1. Research Design

A qualitative descriptive methodology, informed by phenomenological principles [15,16], was employed to explore the lived experiences of pwMND and their caregivers, specifically regarding swallowing and communication impairments. Qualitative description allows for straightforward accounts of participants’ experiences, making it suitable for capturing the everyday realities of those affected. Phenomenological elements were incorporated to deepen the understanding of participants’ subjective experiences, particularly as the research question focused on the lived, embodied impacts of impairment. A constructivist epistemological stance [17] guided the study, recognizing that pwMND and their caregivers’ experiences are shaped by their personal interpretations and social contexts. This manuscript has been prepared in accordance with the Standards for Reporting of Qualitative Research [18].

### 2.2. Ethical Clearance

This study was approved by the relevant institutional ethics committees (approval numbers: HREC/15/QRBW/491 and 2015001814). All participants provided written informed consent prior to taking part in the study.

### 2.3. Recruitment

Potential participants were recruited through two publicly funded hospitals in Queensland, Australia. Due to the small number of people diagnosed with MND in any given year, convenience sampling was utilized to recruit participants for the study. Participants were approached by their treating clinician at the time of an existing appointment and provided with information about the study. If participants consented, their contact details were provided to a member of the research team. Eligible participants were adults with suspected or confirmed diagnosis of MND, who had self-reported communication and swallowing changes associated with their MND diagnosis, or were the informal caregivers of an adult with suspected or confirmed diagnosis of MND, with self-reported communication and swallowing changes related to the MND diagnosis.

### 2.4. Data Collection

The collection of demographic information and qualitative interviews was conducted by a female research assistant, a qualified speech pathologist (BSpPath [Hons]), not known to the participants, who had experience in conducting qualitative interviews. The interview guide was developed following a review of the research literature and applying the clinical experience and knowledge of the research team. The interview guides were administered with flexibility to allow the inclusion, omission, or elaboration of questions as they developed, which allowed a more naturalistic, conversational style interview [17]. Interviews were conducted over the phone at a time convenient to the participant. Each participant was interviewed individually and at one time point only. All participants used verbal communication to participate. One pwMND used a combination of verbal communication and augmentative and alternative communication (AAC) and required support from a speech pathologist known to the participant but not known to the researchers to participate. Real-time member checking (i.e., reflecting back what the participant had said to check accuracy in real time) was utilized throughout interviews. Participants were asked to comment on whether this interpretation was correct and were provided with opportunities to clarify or change their response based on the interviewer’s interpretations. Peer debriefing was utilized throughout the interview and analysis process between the interviewer and members of the research team [RP, AR]. All interviews were audio-recorded with permission from participants and transcribed verbatim following the interview.

### 2.5. Data Analysis

Reflexive thematic analysis, as described by Braun and Clarke [19,20], was utilized to report on the interpretation of the interview transcripts. Analysis was performed with the assistance of NVivo14 software. The first author (RP) took an inductive approach during the analysis, whereby codes were derived directly from the data without reference to a pre-existing theoretical framework. However, the researchers focused on the interpretation of the lived experience when coding the data. Codes were examined to identify relationships and common concepts that were merged. Codes were then re-examined to identify relationships between categories and develop overarching themes. Themes were reviewed against respective coded extracts and the entire data set to ensure that the themes accurately reflected the meanings conveyed by participants. Categories and themes were reviewed by another member of the research team (AR, AF) to ensure agreement with the interpretation of the data and to achieve consensus amongst the researchers.

## 3. Results

A total of 12 participants took part in this study. This comprised six pwMND, all of whom had a confirmed MND diagnosis and self-reported communication and swallowing changes, and six caregivers. All participant dyads were in a spousal relationship. Demographic details for participants are reported in Table 1. Three themes were developed from the qualitative interviews, and where possible, these have been developed specifically in relation to the impact of communication and swallowing changes. However, it is acknowledged that all participants were unable to fully separate the experience of communication and swallowing changes from other physical, emotional, and psychosocial aspects of MND. The themes included (1) “This is the way things are”; (2) Your whole life changes, but some things stay the same; and (3) Juxtaposition to information and support.

### 3.1. Theme 1: “This Is the Way Things Are”

Both pwMND and caregivers spoke about the physical changes to swallowing, speech, and voice, which had varying degrees of impact on mealtimes and communication. All described the progression of symptoms occurring over months and sometimes years, with variability in the rate of decline in function. Some pwMND denied experiencing any difficulties swallowing but then discussed changes to taste and smell, needing to cut food into smaller pieces, needing to eat smaller meals due to fatigue, avoiding certain foods, and food going down the wrong way. For example, one participant stated: *“I don’t have a problem swallowing but occasionally I might choke on something … you know somethings gone down the wrong way.”* (P008). All pwMND reported some changes to their eating, drinking, and mealtime experience. Communication impairments were mostly described in terms of vocal loudness—*“it just seems like my voice box isn’t working as well as it used to”* (P007)—vocal fatigue, and reduced speech intelligibility. Shortness of breath and fatigue levels impacted both swallowing and communication across the participant group.

Importantly, participation in conversations and mealtimes was not just related to speech, voice, and swallowing function. Mobility and positioning were also frequently impacted, where pwMND were able to eat meals, and whether they were able to self-feed: *“there’s other things [positioning and use of hands] … that might stop me from sitting around a table”* (P009). Some participants spoke about different adaptive equipment that facilitated successful participation in mealtimes: *“my fingers are sort of bent up and some stick out a bit … [I’ve] got special spoons and forks where they’ve got a thick handle and a strap that goes over me hand.”* (P006). Similarly, mobility and positioning were also important factors for participants' communication in conversations:

*“what I find though is, when you’re in, when you’re talking to someone, this isn’t about communication difficulties, but if you’re sitting in a wheelchair, people stand about shoulder stand to the stand at the back of you. You’re always twisting your head around looking up at them, they never come and stand in front of you, they stand at the side of you.”*

(P007)

When talking about the physical challenges and impacts on mealtimes and communication associated with MND, all participants spoke about how *“it’s just life, there’s not much we can do about it, so we just gotta get on with it really.”* (P008). This was not to downplay the realities of day-to-day life but rather a radical acceptance of reality and the necessity to take the disease as it comes. Some caregiver participants discussed how this was easier to accept some days than others, and they acknowledged how challenging an MND diagnosis could be: *“I can really well handle it at the moment but then other days it just goes a bit you know not as good.”* (C004).

### 3.2. Theme 2: Your Whole Life Changes, but Some Things Stay the Same

The decline in speech, voice, and swallowing function, along with other physical symptoms of MND, impacted the day-to-day activities that pwMND and their caregivers participated in. Though many were still engaged in active lifestyles, a lot more responsibilities were placed on caregivers both within the home and to support the pwMND to participate in family and social activities. Caregivers were the linchpin for pwMND and provided practical, emotional, and advocacy support in all facets of life. Whilst all pwMND were grateful for the support of their caregivers, the change in role was also a challenge as described by one participant: *“she [caregiver] doesn’t complain but I’m sure it gets to her at times because it’s quite a big burden …she does it but you know she has trouble keeping up … it’s quite difficult”* (P009). Caregivers spoke about the numerous roles they needed to take on to support successful mealtimes. This included thinking ahead about meals, meal preparation, positioning for meals, vigilance around coughing/choking, cutting up food, and cleaning up after meals. For example, one caregiver described her process in meal preparation: *“[I] make sure that the portion sizes are that he doesn’t have to cut it up. If I’m making a curry or something, for example, I wouldn’t make the pieces too large that it needs to be cut.”* (C009). Similarly, caregivers played an important role in facilitating communication not only between the caregiver and the pwMND but also between the pwMND and other people:

*“if you’re talking in a group of people and I’m conversing with somebody … and you’re in a conversation about something and she [pwMND] may be communicating … with somebody else at the time, I may not always notice immediately that she’s having an issue but you do need to develop that second sense of awareness to make sure that she’s in a position where she can communicate adequately and in a stress-free manner you know in a stress-free environment.”*

(C007)

In addition, social lives, work lives, and hobbies changed, though they did not always stop. One participant, who had moved into a residential aged-care service, found new activities to take part in: *“some of us go to bingo and some of us go to the concerts”* (P006). Another participant, whose communication was supported through AAC alongside speech, spoke about the impact of his dysarthria on his ability to take part in Men’s Shed: *“I used to be [secretary] of the men’s shed … I cannot communicate with the [unintelligible] people in the shed.”* (P010). The impact on social activities extended to caregivers, with one caregiver reporting how going out was becoming too difficult: *“I think it’s got to the point where it’s easier if say … we’re having coffee with someone in the morning on the back patio or something.”* (C010). Further to this, another caregiver discussed that while friends were supportive and would come to visit, this often required more effort on the part of the caregiver: *“they’d [friends] like to come and visit and, they stay for about four or six hours, and it’s like they’re here for morning tea, they’re here for lunch …that just creates extra work for me.”* (C008). Some participants discussed how they could no longer travel due to concerns about swallowing: *“we couldn’t go overseas anymore because you don’t know what’s going to happen with his swallowing”* (C010).

PwMND and caregivers spoke about how carers had to assist them to navigate complex health systems, funding schemes, and medical information. Despite the significant life changes, most participants, both pwMND and caregivers, spoke about how many existing relationships and friendships remained the same with family—*“we have a good family base”*— and friends: *“we have friends that perhaps come here … for an afternoon drink or something, who we’ve known for such a long while that they’re understanding the difficulty of what’s happening”* (C010). Similarly, despite the numerous role shifts and additional responsibilities of caregivers, all participants did not believe that this had impacted the dynamic between the pwMND and the carer. One pwMND shared: *“it’s always really good relationship between me and my wife. I mean that’s the whole thing, there were no changes necessary really. What she’s doing for me, I would have done for her too.”* (P004). This was echoed by caregivers as described by one participant who stated: *“I stuck by her and I’m quite happy to look after her”* (C009).

### 3.3. Theme 3: “I Don’t Want to Know Too Much, but I Don’t Want to Know Too Little”: Juxtaposition of Information and Support

Mixed perspectives were presented regarding the level of understanding of what might be expected in the progression of swallowing and speech. Some participants were quite aware of what to expect—*“I expect there to be changes in the communication and with swallowing and um, I’ll eventually get my feed through a tube.”* (P006)—whereas others were not: *“No I haven’t thought about the future, what’s going to happen. That my eating habits going to change quickly I don’t know, I can’t tell you anything about that.”* (P004). PwMND and caregivers had a complex relationship with healthcare information and peer support in relation to MND signs, symptoms, progression, and impact. For caregivers, however, it was essential to know what to expect: *“I want to understand what’s happening and how we’re going to cope with it if his swallowing gets worse. That’s what I need, I need to know those details.”* (C010). Despite this, caregivers also emphasized the need to take the disease as it came: *“we’ll adjust to it and we take it the way it comes one day to the other day.”* (C004).

This was echoed by some pwMND. One participant said: *“I try to just be aware and if things pop up then I know which way to go without knowing all the answers.”* (P009). Some participants conducted their own research to identify if their symptoms were related to MND: *“I looked up the internet when I realized that maybe my voice box, well it’s a muscle and maybe it’s going and, it did say in the MND that, some people can get hoarse.”* (P007). Across the groups, participants had varying degrees of need or desire for the type of information they wanted, the amount of information, and how far ahead they wanted to know. For example, one participant described wanting to read about various aspects of MND but finding the information overwhelming: *“I find these websites and every now and then I go back to them. You read something and you stop because it starts getting to you, so you think I won’t read any more of that.”* (P009).

This was similar to the range of experiences and perceptions of peer support groups. Some participants had attended support groups with other people with neurodegenerative conditions and found that *“It’s good to have these conversations [with peers]. I mean, that you can talk with other people having the same problems and everyone experiences that a different way.”* (P004). Not all participants, however, were open to peer support and were hesitant about meeting other pwMND: *“I haven’t gone out of my way to meet other people with MND. I’m not sure I wouldn’t find that depressing.”* (P009). A juxtaposition was evident across the interviews between wanting just enough information about what to expect and how to manage it, along with an aversion to having too much information. Participants wanted answers but were hesitant about what they would be told or what they would find.

Finally, participants discussed perspectives on accessing support from health professionals. One caregiver summed up the experience of many, stating: *“the issue is knowing who you can see to access …what’s available … and how you access it”* (C007). Only some participants reported accessing a speech pathologist. For example, one participant spoke about their experience of the speech pathologist providing information about future strategies: *“I’ve been to the speech therapist twice now and she explained to me the process of swallowing and what things I could do to keep my mouth clear … she gave me some strategies but they’re not things that I actually need now.”* (P009). In addition, the participant spoke about the discussion with the speech pathologist about their future plans for communication: *“we talked about a speech bank and she’s [speech pathologist] going to find out some information … she explained to me all the different methods that you can communicate.”* (P009). Most caregivers did not speak about accessing health professionals and figured out strategies and adjustments to dysphagia and dysarthria with the pwMND. One caregiver did not yet believe there was a need for these services despite the pwMND experiencing both dysphagia and dysarthria: *“meal options and ways of overcoming that issue [dysphagia] we’ll investigate in more detail with some professional advice as we need to.”* (C007).

## 4. Discussion

The results of the current study demonstrate that the impacts of swallowing and speech changes in MND are wide-ranging and have an impact beyond the physical domains of swallowing and speech. In addition, this study provides new insights into the impacts of swallowing and speech impairments on the lives of caregivers and how caregivers act as essential linchpins for successful mealtimes and communicative interactions. These findings highlight the need for information and support to be family-centered and to recognize caregivers as core members of the multi-disciplinary team.

Consistent with previous research, this study highlights a range of changes to swallowing and speech in MND. Despite discussing necessary changes to food textures and instances of coughing involving food and liquids, some participants denied the presence of dysphagia. This is consistent with the findings from Lisiecka and colleagues [11] who found that participants with difficulties associated with eating and drinking did not consider themselves to have dysphagia. In addition, participants in this study discussed additional functional difficulties with positioning and hand mobility as having a heightened impact on mealtimes. Caregivers were also frequently included as important facilitators of successful mealtimes. These findings highlight the need to move away from symptom-based measures of swallowing and consider a broader assessment of mealtime participation to accurately capture the skills and abilities of pwMND and to assess the way in which caregivers act as important facilitators. Lisiecka and colleagues, however, caution against an overreliance on patient-reported outcome measures due to some pwMND denying or underreporting the severity of symptoms [11]. There is a need for a more holistic, clinician-rated assessment that more accurately captures functioning and participation at mealtimes.

The need to consider dysarthria as only one aspect of communicative function emerged as an important finding in the current study. This is consistent with previous qualitative research in a mixed cohort of people with acquired dysarthria who similarly reported the impact of coexisting functional impairments that further exacerbated communication impairment [21]. In the current study, participants reported numerous barriers to communication participation, including listener barriers, positioning barriers, mobility barriers, and environmental barriers. In their perspective piece, Page and Yorkston [22] argue that communicative participation should be the primary focus for assessment of people with dysarthria. The practice patterns of speech pathologists in the assessment of progressive dysarthria have been explored by Collis and Bloch, who reported that whilst speech pathologists valued targeting impairment, activity, and participation in their management, the clinical reality was that the assessment of progressive dysarthria was predominantly impairment-based [23]. Research regarding non-progressive dysarthria has highlighted inconsistencies in the use of assessment tools, and taken together, these studies have called for the establishment of a dysarthria outcome set [24,25,26]. Work is currently underway to establish a core outcome set for dysarthria after stroke [27], but to the authors’ knowledge, this has not yet been explored relating to dysarthria in progressive conditions such as MND.

This study identifies a nuanced interplay between the provision of information and emotional support in the context of MND care. PwMND and their caregivers in this study reported a range of perspectives regarding seeking sufficient knowledge to make informed decisions about care, plans for the future, and maintain a sense of control amidst uncertainty. These findings echo the results of O’Brien [28], who reported that pwMND utilize three distinct information-seeking behaviors, including active seekers who acquire information from a variety of sources (e.g., health professionals, internet searching, and peer support), selective seekers, who rely on buffers to filter out unsuitable material, and information avoiders. The current study demonstrates that MND caregivers also participated in these information-seeking behaviors. Whilst research and clinical guidelines provide recommendations regarding the communication of information at diagnosis, further research is needed to identify appropriate timing or markers to provide additional information and education to both pwMND and caregivers throughout the course of the disease.

Whilst this study provides important insights into the lived experience of dysphagia and dysarthria from the point of view of pwMND and their carers, some limitations are acknowledged. Interviews were conducted with pwMND and caregivers at a single time point. It would be valuable to conduct further longitudinal qualitative studies that explore the experience of dysphagia and dysarthria over the trajectory of the disease. The majority of participants in this study were still mostly intelligible, and cognition was not assessed. Therefore, there is a need to explore the perspectives of pwMND and caregivers who are using non-verbal communication methods and who may be experiencing cognitive impairments. Finally, all pwMND in this study had a supportive partner as their primary caregiver. It is important to explore the perspectives of pwMND who do not have a spouse as their primary caregiver.

## 5. Conclusions

This qualitative study highlights the complex interplay of functional impairments, participation restrictions, and environmental barriers that influence mealtimes and communicative interactions for pwMND. The current data confirms the need for more holistic assessments that prioritize participation in both meals and communicative situations. Furthermore, there is a need to identify the information and educational needs of pwMND and caregivers throughout the disease trajectory to address not only the physical changes but also the associated psychosocial consequences of dysphagia and dysarthria.

## Figures and Tables

**Table 1:** Participant demographics (n = 12).

Variable		pwMND	Caregivers
Age (years)	<65	2	3
	>65	4	3
Gender	Female	2	4
	Male	4	2
Employment status	Retired/Not working	6	5
	Employed	0	1
Children	Has children	6	6
	Does not have children	0	0
Living arrangement	Home	5	6
	Residential aged-care	1	0
MND onset	Bulbar	1	
	Spinal	5	
Time since diagnosis	<6 months	2	
	12–24 months	3	
	>24 months	1	
Communication method	Verbal	6	
	AAC ^1^	1	
Diet type	Regular	0	
	Modified	6	
	Non-oral	0	

^1^ AAC = augmentative and alternative communication.

## Data Availability

The data presented in this study are not publicly available due to ethical restrictions and to protect the privacy of the participants. Access to the data is restricted by the Royal Brisbane and Women’s Hospital and The University of Queensland in accordance with approved ethical protocols. Interview guides are available upon request.

## Abbreviations

The following abbreviations are used in this manuscript:

MND	Motor neuron disease
pwMND	Person/people with motor neuron disease
